# Hepatotoxicity induced by trastuzumab used for breast cancer adjuvant therapy: a case report

**DOI:** 10.1186/1752-1947-8-417

**Published:** 2014-12-10

**Authors:** Kazuo Ishizuna, Jun Ninomiya, Toshihisa Ogawa, Eiichi Tsuji

**Affiliations:** 3 Breast Center, Dokkyo Medical University Koshigaya Hospital, 2-1-50 Minami-Koshigaya, Koshigaya, Saitama, 343-8555 Japan; 4 Ninomiya Hospital, 491-6 Shin-eicho, Soka, Saitama, 340-0056 Japan

**Keywords:** Breast cancer, Hepatotoxicity, Trastuzumab

## Abstract

**Introduction:**

Trastuzumab is generally considered a highly safe drug, but there have been cases of infusion reaction and cardiotoxicity. This report will present a rare case of hepatotoxicity induced by trastuzumab used for adjuvant therapy of human epidermal growth factor receptor type 2-positive breast cancer.

**Case presentation:**

The patient was a 60-year-old Japanese postmenopausal woman with a non-contributory past medical history. She presented for detailed examination of an abnormality in her left breast. She had left breast cancer (T2N1M0, stage IIB) that was positive for estrogen receptor and progesterone receptor and was human epidermal growth factor receptor type 2 3+. She began receiving epirubicin and cyclophosphamide therapy but developed hepatotoxicity (aspartate aminotransferase 43U/L, alanine aminotransferase 104U/L, alkaline phosphatase 634U/L, and γ-glutamyl transpeptidase 383U/L). Thus, the therapy was discontinued after two cycles, and a weekly paclitaxel therapy was begun. After the absence of an adverse event was confirmed, she also began receiving trastuzumab (4mg/kg) at the second cycle. However, hepatotoxicity (aspartate aminotransferase 267U/L, alanine aminotransferase 246U/L, alkaline phosphatase 553U/L, and γ-glutamyl transpeptidase 240U/L) developed again, and trastuzumab was discontinued. She received paclitaxel monotherapy for a total of four cycles and subsequently underwent partial mastectomy and axillary dissection. After completing adjuvant radiation therapy (breast, 50Gy), she received trastuzumab administration (4mg/kg) but hepatotoxicity (aspartate aminotransferase 47U/L, alanine aminotransferase 102U/L, alkaline phosphatase 377U/L, and γ-glutamyl transpeptidase 91U/L) recurred. Thus, it was discontinued again. There was no hepatitis B or C virus infection, and a drug-induced lymphocyte stimulation test revealed a positive reaction to trastuzumab (stimulation index: 227%). Thereafter she has used only oral letrozole (2.5mg/day) and no recurrent cancer has been observed.

**Conclusions:**

Although trastuzumab is a highly safe drug, one must be mindful of its risk for hepatotoxicity. Periodic monitoring of liver functions is necessary during trastuzumab therapy.

## Introduction

Trastuzumab is generally considered a highly safe drug, but there have been cases of infusion reaction and cardiotoxicity [1–4]. We report here a rare case of hepatotoxicity induced by trastuzumab used for adjuvant therapy of human epidermal growth factor receptor type 2 (HER2)-positive breast cancer and we also include a brief literature review.

## Case presentation

**Figure 1:**
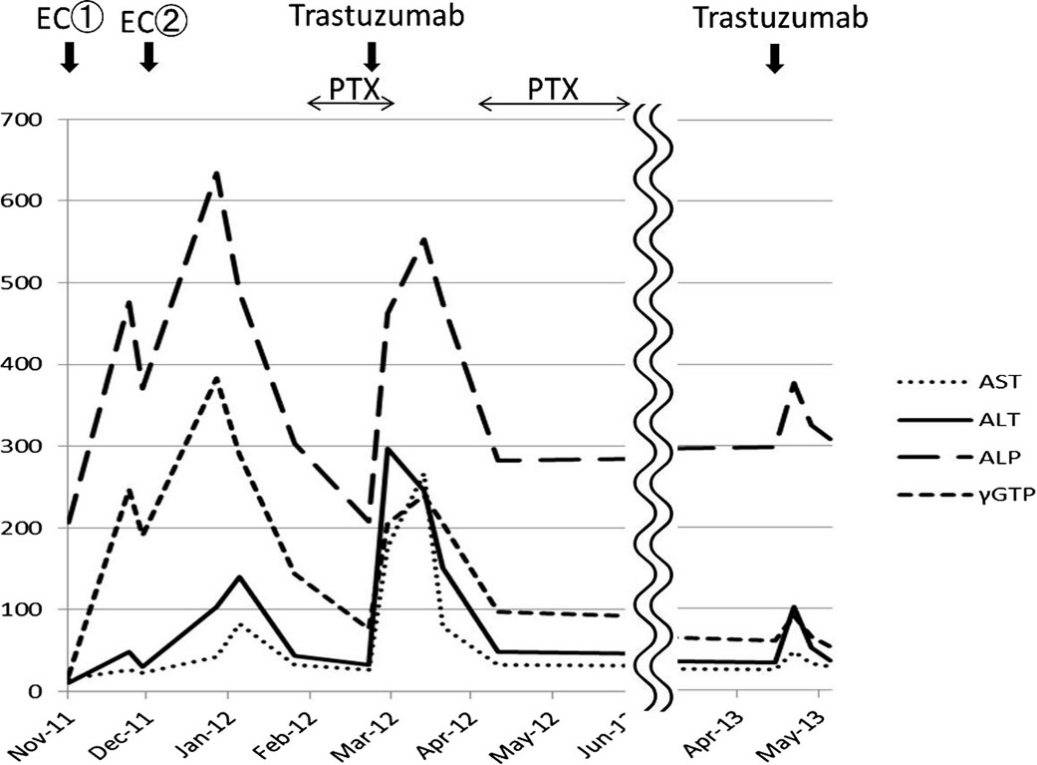
**Treatment course and changes in hepatotoxicity.** The patient was diagnosed with trastuzumab-induced hepatotoxicity. This diagnosis was based on the finding that hepatotoxicity developed not only for a combination of trastuzumab and PTX therapy but also for trastuzumab monotherapy. Abbreviations: ALP, alkaline phosphatase (IU/L); ALT, alanine aminotransferase (IU/L); AST, aspartate aminotransferase (IU/L); EC, epirubicin + cyclophosphamide; γ-GTP, γ glutamyl transpeptidase (IU/L); PTX, paclitaxel.

The patient was a 60-year-old postmenopausal Japanese woman with non-contributory past medical history, alcohol use at a social drinking level, and no history of cigarette smoking. An abnormality was found in her left breast during a physical examination at another hospital, and she was referred and presented to our hospital for a detailed examination in October 2011. The results of the examination revealed left breast cancer (T2N1M0, stage IIB) that was positive for estrogen receptor and progesterone receptor and was HER2 3+. The treatment plan was neoadjuvant chemotherapy. In November, she began receiving epirubicin and cyclophosphamide therapy (epirubicin 75mg/m^2^, cyclophosphamide 600mg/m^2^, day 1 every 3 weeks) but developed hepatotoxicity: aspartate aminotransferase (AST) 43U/L (Grade 1), alanine aminotransferase (ALT) 104U/L (Grade 1), alkaline phosphatase (ALP) 634U/L (Grade 1), and glutamyl transpeptidase (γ-GTP) 383U/L (Grade 3). Thus, the therapy was discontinued after two cycles, and a weekly paclitaxel (PTX) therapy (80mg/m^2^ on days 1, 8, and 15; every 4 weeks) was begun. After the absence of adverse events was confirmed, she also began receiving trastuzumab at a loading dose (4mg/kg) beginning with the second cycle. However, hepatotoxicity (AST 267U/L, Grade 3; ALT 246U/L, Grade 3; ALP 553U/L, Grade 1; and γ-GTP 240U/L, Grade 3) developed again, and trastuzumab was discontinued. There was no elevated bilirubin or abnormality in coagulation tests. After liver functions improved, she received PTX monotherapy for a total of four cycles. In August 2012, she underwent partial mastectomy and axillary dissection, and the pathological diagnosis was papillotubular carcinoma with a depth of invasion of 1.2cm, n-, ly0, v0, negative margin, and histological response to chemotherapy of grade 1b. After completing adjuvant radiation therapy (breast, 50Gy), she was readministered trastuzumab (4mg/kg) beginning in May 2013, but hepatotoxicity (AST 47U/L, Grade 1; ALT 102U/L, Grade 1; ALP 377U/L, Grade 1; and γ-GTP 91U/L, Grade 2) recurred. Thus, it was discontinued again (Figure 1). She was negative for hepatitis C virus antibody and hepatitis B virus DNA, and there was no hepatitis B or C virus infection. A drug-induced lymphocyte stimulation test (DLST) revealed a positive reaction to trastuzumab (stimulation index: 227%). Thereafter she has used only oral letrozole (2.5mg/day) and no recurrent cancer has been observed as of May 2014.

## Discussion

Overexpression of HER2 protein occurs in 20 to 30% of breast cancers and is considered a predictor of poor prognosis [5, 6]. The prognosis of patients with such cancer has improved greatly with the emergence of the molecularly targeted drug trastuzumab [7, 8]. Therefore, it is presently used for advanced and metastatic breast cancer and is also used frequently for adjuvant therapy. It is a major drug for treatment of HER2-positive breast cancer.

The most significant adverse events of trastuzumab are serious infusion reactions and cardiotoxicity [1–4]. Other serious adverse events are rare in general clinical practice. In particular, trastuzumab-induced hepatotoxicity is rare and there are only three past reports in the English literature. These reports are described below.

In one report, Muñoz *et al*. presented a case of HER2-positive breast cancer that underwent surgery and adjuvant trastuzumab therapy (8mg/kg). The patient developed hepatotoxicity (AST, Grade 3; ALT, Grade 4), but liver function tests showed normal values 1 month after trastuzumab was discontinued. Therefore, trastuzumab was reintroduced at 2mg/kg every week and she completed 1 year of treatment without recurrence of hepatotoxicity. This patient had received fluorouracil, doxorubicin, and cyclophosphamide therapy up to 1 month before trastuzumab was begun and had hepatotoxicity (AST, Grade 3; ALT, Grade 4). These authors discussed that trastuzumab probably caused hepatotoxicity in a dose-dependent manner, but chemotherapy-induced hepatotoxicity also probably played a role in trastuzumab-induced hepatotoxicity [9].

In another report, Srinivasan *et al*. presented a case of HER2-positive breast cancer that underwent neoadjuvant therapy (weekly PTX and trastuzumab). Consequently, the patient developed hepatotoxicity (AST and ALT, Grade 3; ALP, Grade 2). When trastuzumab was reintroduced as monotherapy after surgery, she once again developed hepatotoxicity (AST and ALT, Grade 3; ALP, Grade 2) [10].

In still another report, Vucicevic *et al*. reported on a case of HER2-positive breast cancer in which adjuvant trastuzumab monotherapy was used. The patient developed hepatotoxicity 6 months after the first administration. In this patient, the transaminase level continued to elevate for 2 months after trastuzumab was discontinued (AST and ALT, Grade 3; ALP, Grade 2), but the results of her liver function tests were normal on the fourth month. These authors thought that the long half-life of trastuzumab was a cause of prolonged hepatotoxicity [11].

Our patient did not have a family history of liver disease, was not taking any drug regularly, and had no hepatitis B or C virus infection. She was diagnosed with trastuzumab-induced hepatotoxicity. This diagnosis was based on the finding that hepatotoxicity developed not only for a combination of trastuzumab and PTX therapy but also for trastuzumab monotherapy and that the DLST revealed a positive reaction to trastuzumab.

Our patient is the fourth reported case of trastuzumab-induced hepatotoxicity based on our literature search and the first case in which trastuzumab was identified as the causative drug using DLST. Two of the previous reported cases had developed hepatotoxicity after the first administration of trastuzumab. However, one case had developed hepatotoxicity a while after beginning trastuzumab therapy and, therefore, caution is required. When trastuzumab-induced hepatotoxicity is detected early, liver dysfunction can be reversed and more serious complications can be prevented. Thus, periodic liver function tests are thought to be necessary during trastuzumab therapy.

## Conclusions

Although trastuzumab is highly safe, one must be mindful of its risk for hepatotoxicity and periodically perform liver function tests during trastuzumab therapy.

## Consent

Written informed consent was obtained from the patient for publication of this manuscript and any accompanying images. A copy of the written consent is available for review by the Editor-in-Chief of this journal.

## Abbreviations

ALP: Alkaline phosphatase
ALT: Alanine aminotransferase
AST: Aspartate aminotransferase
DLST: Drug-induced lymphocyte stimulation test
γ-GTP: γ-glutamyl transpeptidase
HER2: Human epidermal growth factor receptor type 2
PTX: Paclitaxel.
